# Diagnostic Value of Neutrophil‐to‐Lymphocyte Ratio and Mean Platelet Volume as Predictors of Liver Fibrosis Severity in MASLD: A Cross‐Sectional Study

**DOI:** 10.1002/hsr2.72447

**Published:** 2026-04-28

**Authors:** Ghannei Olfa, Olfa Lataoui, Sefrihi Fatima Ezzahra, Ben Amor Soumaya, Trimech Mayada

**Affiliations:** ^1^ Department of Gastroenterology University Hospital Tahar Sfar Mahdia Tunisia

**Keywords:** liver fibrosis, mean platelet volume (MPV), metabolic dysfunction‐associated steatotic liver disease (MASLD), neutrophil‐to‐lymphocyte ratio (NLR), non‐invasive biomarkers, transient elastography

## Abstract

**Introduction:**

Metabolic dysfunction‐associated steatotic liver disease (MASLD) has become a leading cause of chronic liver disease worldwide, yet simple and reliable non‐invasive markers for fibrosis assessment remain limited. Accessible blood‐based indicators could aid early detection of significant fibrosis. This cross‐sectional study evaluated the diagnostic performance of neutrophil‐to‐lymphocyte ratio (NLR) and mean platelet volume (MPV) as non‐invasive predictors of liver fibrosis severity in patients with metabolic dysfunction‐associated steatotic liver disease (MASLD).

**Methods:**

This cross‐sectional study analyzed data extracted from medical records collected between 2020 and 2023. MASLD diagnosis was based on clinical, laboratory, and imaging findings. Liver fibrosis stage was assessed using transient elastography. Statistical analyses included comparative tests between fibrosis groups and ROC curve analysis to identify optimal cut‐off values and evaluate diagnostic performance.

**Results:**

A total of 140 patients were included. Patients were divided into two groups: non‐significant fibrosis (F < F2; *n* = 95, 68.6%) and significant fibrosis (F ≥ F2; *n* = 45, 31.4%). Mean NLR was 1.77 in F < F2 versus 2.57 in F ≥ F2 (*p *< 0.0001). ROC analysis identified an optimal NLR cut‐off of 1.79, showing 90% sensitivity, 68.7% specificity, and an AUC of 0.812 (95% CI 0.74–0.88). Mean MPV was 8.84 fL in F < F2 versus 9.95 fL in F ≥ F2 (*p *< 0.0001). The optimal MPV threshold was 9.05 fL, yielding 66% sensitivity, 65.7% specificity, and an AUC of 0.71 (95% CI: 0.62–0.81). NLR demonstrated excellent accuracy for identifying significant fibrosis, while MPV showed moderate discriminatory ability.

**Conclusion:**

NLR and MPV correlate significantly with fibrosis severity and may serve as accessible, low‐cost adjunctive biomarkers for early risk stratification of MASLD patients, especially in primary care or resource‐limited settings.

## 1 Introduction

Over the past two decades, Metabolic Dysfunction‐Associated Steatotic Liver Disease (MASLD) has become the most prevalent chronic liver condition worldwide, affecting approximately one‐quarter of the adult population [1]. The global rise in MASLD parallels the obesity epidemic and is strongly associated with metabolic syndrome and its components [2, 3]. MASLD‐related cirrhosis and hepatocellular carcinoma (HCC) are steadily increasing, with MASLD projected to become a leading cause of liver transplantation [4]. The burden of MASLD is particularly high in the Middle East and North Africa (MENA) region, where prevalence estimates exceed 35% in the general population and are even higher among individuals with obesity and type 2 diabetes. In North Africa, although detailed country‐specific prevalence data remain limited, available evidence indicates a rapidly increasing burden driven by epidemiological transitions, including rising rates of obesity, diabetes, and metabolic syndrome [5]. These trends highlight the growing public health importance of MASLD in countries such as Tunisia.

Fibrosis stage is the most powerful prognostic determinant in MASLD, justifying its assessment for morbidity prediction, risk stratification, and follow‐up planning [2]. Liver biopsy, historically considered the reference standard, has major limitations including invasiveness, sampling variability, procedural risk, and cost [6]. Consequently, non‐invasive methods such as transient elastography (TE) with FibroScan have been increasingly adopted to assess liver stiffness and predict complications [7].

Despite its advantages, TE is unavailable in many hospitals, particularly in smaller or remote centers, highlighting the need for accessible, low‐cost biomarkers [8]. Chronic systemic inflammation drives progression from steatosis to Metabolic‐Associated Steatohepatitis (MASH) and fibrosis, mediated by immune‐induced hepatic injury, oxidative stress, and metabolic dysregulation. The neutrophil‐to‐lymphocyte ratio (NLR), derived from routine blood counts, is associated with histological inflammation and fibrosis in MASLD and has proven prognostic value in other systemic diseases. Similarly, mean platelet volume (MPV), reflecting platelet activation, correlates with inflammation and fibrosis in chronic liver disease.

Because both NLR and MPV are inexpensive and widely available, they may support early fibrosis risk stratification and guide monitoring or referral, especially where TE is not accessible. This study investigates the association of NLR and MPV with hepatic steatosis and fibrosis severity, as measured by TE and the controlled attenuation parameter (CAP) [9].

## 2 Methods

### 2.1 Study Design

We conducted a retrospective cross‐sectional study based on primary data extracted from the medical records of patients with MASLD who were managed at our hospital between 2020 and 2023. Laboratory biomarkers and TE with CAP measurements were retrieved from the same clinical records.

### 2.2 Ethical Considerations

This study was approved by the hospital ethics committee (CEM_2024_01_02). Given the retrospective design and the use of fully anonymized data extracted from medical records, the requirement for informed consent was formally waived by the ethics committee.

### 2.3 Sample Size Justification

Sample size was calculated for receiver operating characteristic (ROC) curve analysis assuming a null hypothesis AUC (AUC₀) of 0.60, representing the minimal clinically acceptable discriminatory ability beyond chance (AUC = 0.50), and an expected AUC (AUC₁) of 0.80 based on prior literature. Assuming a 1:2 ratio between patients with and without significant fibrosis, a two‐sided *α* of 0.05, and 80% power, the required sample size was achieved within the study period. All eligible patients meeting the inclusion criteria during the study timeframe were included in the final analysis.

### 2.4 Patient Selection

A consecutive sampling technique was used. Eligible patients were adults ( ≥ 18 years) with confirmed MASLD and valid TE/CAP assessments performed at the Hepatology Unit of Mahdia University Hospital between January 2020 and December 2023. MASLD diagnosis required imaging evidence of hepatic steatosis—initially screened via ultrasound or CT/MRI and quantified using CAP—together with at least one cardiometabolic risk factor, including elevated BMI, prediabetes/diabetes, hypertension, hypertriglyceridemia, or low HDL‐C, in accordance with the 2023 international MASLD consensus definition. Exclusion criteria included chronic liver disease of other etiologies (viral hepatitis, autoimmune liver disease, alcoholic liver disease, cholestatic disorders), hematologic disorders, active malignancy, systemic infection, or recent use of immunosuppressive therapy, chemotherapy, or corticosteroids within the preceding 3 months. Patients were classified under the MASLD category according to the 2023 international consensus definition. Only individuals fulfilling the diagnostic criteria for MASLD were included in the study. Patients with significant alcohol consumption or other chronic liver diseases were excluded to avoid misclassification within the broader SLD spectrum.

Sub‐classification within the SLD spectrum (e.g., MetALD or other SLD categories) was not separately analyzed, as the study objective focused specifically on fibrosis severity in patients meeting MASLD criteria. Liver enzyme distributions (AST and ALT) were analyzed exclusively within this MASLD‐defined cohort.

### 2.5 Data Collection

Variables collected included age, sex, BMI, ALT, AST, HDL‐C, TG, fasting glucose, HbA1c, CRP, leukocyte, neutrophil, lymphocyte, platelet counts, and MPV. BMI was measured using a calibrated digital scale and wall‐mounted stadiometer, with patients wearing light clothing and no footwear. Blood samples were obtained after an overnight fast of at least 8 h. Venous blood was collected on the same day as transient elastography or within a maximum interval of 7 days. Biochemical assays (ALT, AST, lipids, glucose, CRP) were performed in the hospital's certified biochemistry laboratory using a Cobas 6000 analyzer (Roche Diagnostics, Mannheim, Germany) following manufacturer protocols. Hematologic parameters, including MPV, were measured using an automated hematology analyzer (Sysmex XN‐1000, Sysmex Corporation, Kobe, Japan) within 2 h of sample collection. NLR was calculated as the neutrophil count divided by the lymphocyte count.

### 2.6 Fibrosis and Steatosis Assessment

Liver stiffness was assessed using transient elastography (FibroScan 502 Touch, Echosens, Paris, France) by trained operators with at least 100 prior examinations. Measurements were performed after a minimum fasting period of 3 h, using the M or XL probe as appropriate according to the manufacturer's recommendation. A valid examination required at least 10 successful measurements, a success rate ≥ 60%, and an interquartile range/median ≤ 30%. Fibrosis stage, indicated by F (ranging from F0 to F4), was determined using established FibroScan cut‐off values as recommended by EASL guidelines. Significant fibrosis was defined as liver stiffness ≥ 7.0 kPa (F ≥ 2). Moderate‐to‐severe steatosis was defined as CAP ≥ 259 dB/m. Patients were categorized as having non‐significant fibrosis (F < 2) or significant fibrosis (F ≥ 2).

### 2.7 Statistical Analysis

Descriptive statistics summarized clinical characteristics. Normality was tested with Kolmogorov–Smirnov. Pearson or Spearman correlation coefficients were used to assess associations between biomarkers and TE/CAP. ROC analyses were used to evaluate the diagnostic performance of NLR and MPV for significant fibrosis, including AUC, sensitivity, specificity, positive predictive value, and negative predictive value. Multivariate logistic regression was performed to identify independent predictors of significant fibrosis. Variables with a *p* value < 0.20 in univariate analysis were entered into the multivariable logistic regression model. The analysis was adjusted for age, sex, BMI, diabetes, hypertension, and dyslipidemia. Multicollinearity among variables was assessed using variance inflation factors (VIF).

A two‐sided *p* value < 0.05 was considered statistically significant. All analyses were performed using SPSS version 20.0 (IBM Corp., Armonk, NY, USA). Analyses were performed using SPSS version 20.0 (IBM Corp., Armonk, NY, USA).

## 3 Results

### 3.1 Demographics and Laboratory Characteristics

Among the 140 MASLD patients included, the mean age was 53.0 years, ranging from 15 to 78 years. The study population was predominantly female (65.7%), while males accounted for 34.3%. The baseline characteristics are summarized in Table 1. Regarding fibrosis, 95 patients (68.6%) had a stage below F2 with an LSM < 7 kPa, whereas 45 patients (31.4%) had fibrosis ≥ F2. ALT and AST showed non‐normal distributions; however, means and standard deviations are presented to facilitate comparison with previous MASLD studies using similar reporting formats. The rest of the study parameters are presented in Table 2.

**Table 1:** Basic characteristics of research subjects.

Variable	Total (140)
Gender	
Male (%)	48 (34.3)
Female (%)	92 (65.7)
Age, mean in years (SD)	53 years ± 12.6 (15–78) years
BMI, mean in kg/m^2^ (SD)	
BMI ≥ 25 (%)	99 (70.7)
BMI < 25 (%)	41 (29.3)
Diabetes mellitus	
Yes (%)	60 (42.8)
No (%)	80 (57.1)
Metabolic syndrome	
Yes (%)	53 (37.8)
No (%)	87 (62.1)
Fibroscan	
Non‐significant fibrosis (%)	95 (68.6)
Significant fibrosis (%)	45 (31.4%)

**Table 2:** Study characteristics comparing the mild to moderate fibrosis groups.

	Mild fibrosis (0–1)	Advanced/significant fibrosis (2–4)	*p* value
Patients			
Female	67 (72.8%)	25 (27.2%)	0.004
Male	31 (64.6%)	17 (35.4%)	0.312
Age (mean ± SD)	55.74 (13.07)	55.62 (11.57)	0.957
BMI	30.2 (3.62)	37.87 (6.65)	< 0.001
Diabetes mellitus	22 (36.7%)	38 (63.3%)	< 0.001
Dyslipidemia	10 (25.6%)	29 (74.4%)	< 0.001
High blood pressure	26 (70.3%)	11 (29.7%)	0.788
Metabolic syndrome	17 (32.1%)	36 (67.9%)	< 0.001
Mean ± DS			
AST (U/L)	34.03 (35.35)	36.01 (27.41)	0.831
ALT (U/L)	66.20 (29.41)	59.54 (31.09)	0.461
TG (mg/dL)	1.23 (0.47)	3.01 (5.79)	< 0.001
NLR	1.77 (1.25)	2.57 (0.811)	< 0.001
MPV	8.83 (1.40)	9.95 (1.41)	< 0.001
APRI.SCOREmedian (IQR)	0.86 (0.32–1.50)	1.20 (0.90–1.70)	< 0.001

Abbreviations: ALT, alanine transaminase; AST, aspartate transaminase; CRP, C‐reactive protein; MPV, mean platelet volume; NLR, neutrophil‐to‐lymphocyte ratio; TG, triglycerides.

### 3.2 Correlation of NLR and MPV With Fibrosis

**Figure 1:**
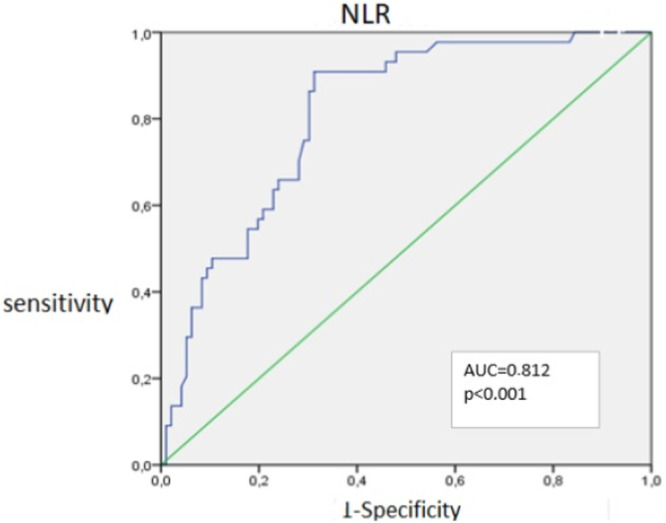
ROC curve analysis of neutrophil‐to‐lymphocyte ratio (NLR) for the detection of significant fibrosis (F ≥ 2) in MASLD patients. The AUC was 0.812 (95% CI: 0.74–0.88; *p* < 0.001), with an optimal cut‐off value of 2.1 (sensitivity 72%, specificity 70%).

Mean NLR: 1.77 (F < F2) versus 2.57 (F ≥ F2), *p*< 0.001. ROC analysis identified an optimal NLR cut‐off 1.79, yielding a sensitivity of 90% and a specificity of 68.7%, with an AUC of 0.812 (95% CI 0.74–0.88), as illustrated in Figure 1. Pearson correlation with CAP: *r* = 0.14, *p* = 0.109. ANOVA confirmed NLR variation across steatosis grades (*p *< 0.001).

**Figure 2:**
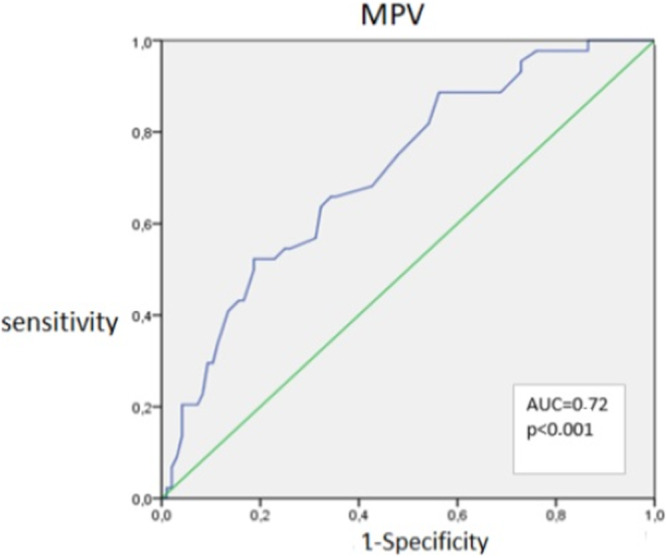
ROC curve of mean platelet volume (MPV) for the detection of significant fibrosis (F ≥ 2); AUC 0.71 (95% CI: 0.62–0.81; *p* < 0.001).

Mean MPV: 8.83 fL (F < F2) versus 9.95 fL (F ≥ F2), *p* < 0.001. ROC cut‐off 9.05 fL: 66% sensitivity, 65.7% specificity, AUC 0.71 (95% CI 0.62–0.81). These results are illustrated in Figure 2.

### 3.3 Multivariate Analysis

In the multivariable logistic regression model adjusted for age, sex, body mass index, diabetes mellitus, hypertension, and dyslipidemia, NLR remained independently associated with significant fibrosis (OR 2.34, 95% CI 1.42–3.89, *p* = 0.001). Body mass index (OR 1.14, 95% CI 1.07–1.22, *p* < 0.001), diabetes mellitus (OR 3.87, 95% CI 1.92–7.81, *p* < 0.001), and dyslipidemia (OR 2.74, 95% CI 1.29–5.82, *p* = 0.008) were also independently associated with significant fibrosis. MPV (OR 1.21, 95% CI 0.89–1.63, *p* = 0.214), age, sex, and hypertension were not independently significant (Table 3).

**Table 3:** Multivariable logistic regression for predictors of significant fibrosis (F ≥ 2).

Variable	Odds ratio (OR)	95% Confidence interval	*p* value
Neutrophil‐to‐lymphocyte ratio (NLR)	2.34	1.42–3.89	0.001
Mean platelet volume (MPV)	1.21	0.89–1.63	0.214
Age (years)	1.01	0.98–1.04	0.42
Male sex	1.18	0.61–2.29	0.61
Body mass index (kg/m²)	1.14	1.07–1.22	< 0.001
Diabetes mellitus	3.87	1.92–7.81	< 0.001
Hypertension	1.09	0.52–2.29	0.82
Dyslipidemia	2.74	1.29–5.82	0.008

## 4 Discussion

### 4.1 Summary of Key Findings

In this cross‐sectional study of 140 MASLD patients, we found that NLR and MPV were significantly associated with the severity of liver fibrosis as measured by TE/CAP. NLR demonstrated good discriminatory ability, while MPV showed moderate performance. NLR showed a weak correlation with hepatic steatosis (CAP), but ANOVA revealed significant variation across steatosis grades. These findings suggest that both NLR and MPV may serve as accessible, non‐invasive biomarkers for identifying patients at risk of advanced fibrosis.

### 4.2 Interpretation and Comparison With Existing Literature

The observed association between elevated NLR and advanced fibrosis aligns with prior studies and meta‐analyses, reinforcing the role of systemic inflammation in MASLD progression [10, 11]. NLR reflects the balance between neutrophil‐mediated inflammation and lymphocyte‐mediated immune regulation; a higher ratio indicates heightened inflammatory activity, which drives hepatic fibrogenesis.

MPV, as a marker of platelet activation, showed moderate predictive ability. Its elevation in patients with significant fibrosis likely reflects the contribution of activated platelets to inflammatory and fibrotic pathways in the liver. Our MPV cut‐off (9.05 fL) is consistent with previous studies by Lauszus et al. [12] and Hanafy et al. [13], confirming the reproducibility of MPV as a potential non‐invasive fibrosis biomarker.

Although Pearson correlation analysis showed only a weak and non‐significant linear association between NLR and CAP values (r = 0.14, *p* = 0.109), categorical comparison across steatosis grades using ANOVA demonstrated significant differences in mean NLR levels. This discrepancy may indicate that the relationship between inflammatory burden and hepatic steatosis is not strictly linear. Rather, NLR may increase across steatosis categories in a threshold‐dependent or non‐linear pattern. These findings underscore the complex interplay between systemic inflammation, hepatic fat accumulation, and fibrogenesis in MASLD.

Our study also confirmed that metabolic comorbidities, particularly type 2 diabetes, dyslipidemia, and metabolic syndrome, were significantly associated with advanced fibrosis. Patients with type 2 diabetes exhibited a higher prevalence of significant fibrosis, consistent with previous reports indicating that diabetes accelerates MASLD progression and increases the risk of fibrosis and HCC [14, 15, 16]. This underscores the importance of aggressive metabolic risk factor management in patients with MASLD.

### 4.3 Clinical Implications

The findings have several clinical implications:

− Early risk stratification: NLR and MPV can serve as low‐cost, readily available biomarkers to identify MASLD patients at high risk for advanced fibrosis, especially in primary care or resource‐limited settings where TE is unavailable.
− Guidance for referral: Patients with elevated NLR or MPV could be prioritized for further imaging, TE, or specialist referral, potentially improving early detection of advanced fibrosis.
− Integration with metabolic management: Monitoring NLR and MPV may help clinicians identify patients who would benefit most from intensive metabolic and lifestyle interventions to slow fibrosis progression.

### 4.4 Strengths of the Study

The study benefits from the inclusion of a well‐characterized MASLD cohort with confirmed TE/CAP measurements, along with the evaluation of both NLR and MPV, which are inexpensive and routinely measured biomarkers. Additionally, the multivariate analysis adjusted for key metabolic comorbidities demonstrates that NLR is independently associated with advanced fibrosis.

### 4.5 Limitations

This study has several limitations, including its cross‐sectional design, which restricts causal inference and prevents evaluation of temporal progression, and its single‐center setting, which may limit the generalizability of the findings. The sample size, while adequate for primary analyses, reduces the ability to explore subgroup effects. Additionally, the use of TE/CAP instead of liver biopsy, although validated, introduces inherent variability compared with histological assessment. Some important confounders, such as alcohol consumption, dietary patterns, genetic predisposition, and medication use, were not measured. Finally, the absence of longitudinal follow‐up limits the ability to determine the predictive value of the biomarkers for clinical outcomes such as cirrhosis or hepatocellular carcinoma. External validation in independent cohorts is required before routine clinical implementation.

## 5 Future Directions

Prospective, multicenter studies with longitudinal follow‐up and liver biopsy correlation are warranted to validate the predictive value of NLR and MPV. Additionally, combining these biomarkers with other non‐invasive fibrosis indices or imaging techniques may improve accuracy and allow integration into clinical risk stratification algorithms.

## 6 Conclusion

In this cohort of MASLD patients, NLR demonstrated good discriminatory performance for significant fibrosis and remained independently associated after adjustment for metabolic confounders. MPV showed moderate accuracy. While these inexpensive and accessible biomarkers may assist early risk stratification in resource‐limited settings, external validation and prospective studies are required before clinical integration.

## Author Contributions

**Ghannei Olfa:** conceptualization, methodology, writing – original draft, and reviewing. **Olfa Lataoui:** methodology, data curation, formal analysis, and supervision. **Sefrihi Fatima Ezzahra:** methodology, data curation, formal analysis, writing – review and editing. **Ben Amor Soumaya:** methodology and formal analysis. **Trimech Mayada:** software and supervision.

## Funding

The authors have nothing to report.

## Conflicts of Interest

The authors declare no conflicts of interest.

## Transparency Statement

The lead author, Ghannei Olfa, affirms that this manuscript is an honest, accurate, and transparent account of the study being reported; that no important aspects of the study have been omitted; and that any discrepancies from the study as planned (and, if relevant, registered) have been explained.

## Data Availability

Data supporting the findings are available within the article and supporting materials.
